# Human Organoids and Organ-on-Chip for Biotoxin Assessment: Applications, Best Practices, and a Translational Roadmap

**DOI:** 10.3390/toxins18030149

**Published:** 2026-03-19

**Authors:** Mingzhu Li, Shuhong Huang, Jinze Jia, Yixing Feng, Jing Zhang

**Affiliations:** 1 Beijing Key Laboratory of Diagnostic and Traceability Technologies for Food Poisoning, Beijing Center for Disease Control and Prevention, Beijing 100013, China; limingzhu90@163.com (M.L.); jiajz13269899611@163.com (J.J.); fengyxioz@126.com (Y.F.); 2 School of Public Health, Capital Medical University, Beijing 100069, China; huangshh1349@163.com

**Keywords:** human organoids, organ-on-chip, enterotoxins, mycotoxins, algal biotoxins

## Abstract

Human organoids and organ-on-chip/microphysiological systems (OoC/MPS) are increasingly used as new-approach methodologies for biotoxin assessment. They retain human-relevant tissue organization and enable interpretable analysis of exposure geometry, barrier transport, perfusion, and (when needed) multi-organ coupling. In this review, we synthesize primary evidence across major toxin classes, including bacterial enterotoxins (e.g., cholera toxin, heat-stable enterotoxins, Shiga toxins), mycotoxins (e.g., aflatoxin B1, ochratoxin A, deoxynivalenol), and algal/cyanobacterial toxins (e.g., saxitoxin, domoic acid, microcystins, biliatresone). We emphasize studies that clearly define toxin identity and exposure context and that demonstrate mechanism-critical model competencies under assay conditions. We highlight decision-informative functional endpoints that align with the dominant pathophysiology. These include cystic fibrosis transmembrane conductance regulator (CFTR)-dependent secretion in human enteroids/colonoids, transporter-linked proximal tubular injury in kidney MPS, gut–kidney axis injury from Shiga toxin-producing *E. coli* in microfluidic systems, and multi-electrode array (MEA) network readouts in human 3D neural tissues. We then summarize best practices that improve cross-study comparability. These include reporting delivered versus nominal exposure, assessing recovery/mass balance and device/material interactions, applying proportional biological qualification (polarity, transporter/enzymatic competence, functional stability), defining a minimal comparable endpoint core, and preserving QIVIVE readiness in reporting. Finally, we outline near-term priorities for the field, including chronic low-dose and mixture designs, harmonized reference panels and acceptance criteria, and fit-for-purpose escalation to coupled OoC/MPS only when perfusion or organ–organ coupling is expected to change the interpretation.

## 1. Introduction

Biotoxins relevant to the food–water–infection continuum include bacterial enterotoxins (e.g., cholera toxin, heat-stable enterotoxin A (STa), Shiga toxins) [1,2,3], fungal mycotoxins (e.g., aflatoxin B1, ochratoxin A, deoxynivalenol) [4], algal/cyanobacterial toxins (e.g., saxitoxin, domoic acid, microcystins) [5,6,7] and plant toxins [5,6]. These toxins together drive outbreaks and can also contribute to chronic health risks under repeated or sustained low-level exposure over time [4,7]. Recent public-health reports underscore the continuing burden of cholera [8], recurrent paralytic shellfish poisoning clusters caused by saxitoxins [9], and the need for guideline-based control of cyanobacterial microcystins in drinking and recreational waters [10]. Their dominant pathophysiology spans secretory diarrhea (enterotoxins) [11], hepatotoxicity (microcystins) [12] and nephrotoxicity (ochratoxin A) [13]. They can also cause acute neurotoxicity with well-defined clinical syndromes such as amnesic shellfish poisoning (domoic acid) and paralytic shellfish poisoning (saxitoxins) [14]. These realities highlight the need for human-relevant test systems that resolve the mechanisms and potency under realistic exposure scenarios.

Legacy test systems have important translational gaps. Immortalized 2D cell lines in monolayer culture rarely recapitulate organ-level vectorial transport, metabolic competence, epithelial polarity, or the cellular heterogeneity that shapes toxicodynamics [15]. However, cell line-based systems are not restricted to 2D monolayers. Three-dimensional cell line spheroids and co-culture systems are also used in toxicological research. These models can recover some tissue-like features, such as cell–cell interactions and multicellular microenvironments [16,17]. They also provide an important bridge between conventional 2D monolayers and more advanced organoid-based systems. Animal models, while invaluable, diverge from humans in enzyme/transporter networks, bioactivation pathways (e.g., aflatoxin B1), and barrier architecture. These differences complicate quantitative translation [15,18]. Beyond scientific limits, the 3Rs (replacement, reduction, refinement) are now mirrored in policy. The United States Food and Drug Administration (FDA) Modernization Act 2.0 removed the statutory animal-test mandate for certain submissions [19]. The FDA has since outlined steps to reduce animal testing in preclinical safety studies and to encourage the inclusion of new approach methodologies (NAMs) data in Investigational New Drug (IND) applications [19]. At the U.S. Environmental Protection Agency (EPA), the December 2021 NAMs Work Plan remained the agency’s formal framework through 2024. The EPA announced in January 2026 a recommitment to the 2035 goal of eliminating mammalian testing requests and funding [20,21]. We also note that the World Health Organization (WHO) has recently highlighted NAMs in the context of future food safety risk assessment [22].

Against this backdrop, human organoids and organ-on-chip (OoC)/microphysiological systems (MPS) are best viewed as a tiered toolkit within advanced in vitro models (alongside spheroids and related 3D constructs) [15]. In this review, we use human organoids to encompass both (i) 3D organoid cultures—self-organized tissues from primary or stem cell sources that retain lineage diversity and epithelial polarity—and (ii) organoid-derived epithelial monolayers (ODMs), which are widely used to enforce and verify polarity and to obtain quantitative barrier and transport readouts under controlled apical versus basolateral dosing [e.g., transepithelial electrical resistance (TEER), tracer flux/apparent permeability coefficient (P_app), and transporter assays] [23]. OoC/MPS adds perfusion and programmable exposure kinetics and can enable organ–organ coupling when systemic distribution is central to the question. However, delivered concentrations may deviate from nominal dosing due to device/material-dependent compound loss, so OoC studies should report delivered concentrations (as well as recovery/mass balance, and free concentration (C_free) where feasible) to support quantitative interpretation [24,25].

Human organoid platforms are already deployed for biotoxin research. Human intestinal organoids/enteroids recapitulate enterotoxin mechanisms. They show cystic fibrosis transmembrane conductance regulator (CFTR)-dependent swelling for cholera toxin pharmacology [26] and guanylyl cyclase C-mediated secretion in response to heat-stable enterotoxins and linaclotide [27]. These features enable mechanism-anchored inhibitor/agonist testing in the human epithelium. An antibiotic-responsive gut–kidney axis on-chip (Caco-2 ↔ HKC-8) enabled simultaneous observation of intestinal O157 infection and Shiga toxin 2 (Stx2)-associated injury in the kidney compartment. In this model, gut→kidney transport was modeled and predicted to be limited (~0.12% at 72 h). Antibiotic choice (ciprofloxacin vs. gentamicin) differentially increased Stx2 release and was accompanied by reduced kidney module TEER/viability [28]. For mycotoxins/plant toxins, a human kidney microphysiological system quantified ochratoxin A nephrotoxicity with primary proximal tubule cells [13]. Human liver organoids exposed to biliatresone reproduced cholangiocyte polarity, tight junctions, and ciliation defects characteristic of biliary atresia-like injury [29]. In the nervous system, human brain organoids and induced pluripotent stem cell (iPSC)-derived 3D brain microphysiological systems equipped with high-density multi-electrode arrays (MEAs) enable functional profiling of algal neurotoxins. In this context, domoic acid was used as a positive control and showed the expected activity profile in the 3D brain MPS-MEA assay. These platforms also showed that saxitoxin exacerbates Zika-induced neuronal death in human brain organoids [30,31]. Together, these findings underscore the value of network physiology readouts alongside morphology and molecular markers.

Building on this evidence, we (i) synthesize what organoid-based formats (3D organoids and organoid-derived monolayers, ODMs) and OoC/MPS already deliver for biotoxin assessment across intestinal, hepatobiliary, renal, and neural systems; (ii) distill best practices in exposure design, dosimetry (nominal vs. C_free), device/material choice, and biological qualification (transporters/enzymes, polarity, barrier and network physiology); and (iii) outline a translational roadmap linking in vitro effect levels to human exposure via quantitative in vitro–in vivo extrapolation (QIVIVE). We anchor this roadmap in Good Cell Culture Practice (GCCP) 2.0 and the OECD Guidance Document on Good In Vitro Method Practices (GIVIMP) to strengthen reproducibility and regulatory readability [32,33].

## 2. Platform Definitions

In this review, we focus on organoid-based formats, including 3D organoids and organoid-derived monolayers (ODMs), as well as organ-on-chip/microphysiological systems (OoC/MPS). Spheroids are included here only for conceptual distinction. They are not a primary focus of this review.

Spheroids are relatively simple 3D multicellular aggregates [34]. They are usually formed under non-adherent or scaffold-free conditions from cell lines, primary cells, or mixed co-cultures [34]. Common spheroid methods include hanging-drop culture, liquid-overlay or low-attachment plates, spinner or rotating suspension culture, microwells, and microfluidic platforms [34]. Compared with organoids, spheroids usually show lower lineage complexity and less organotypic organization [35,36].

By contrast, organoids are self-organized 3D tissues that arise from pluripotent or adult stem/progenitor cells and recapitulate the key structural and functional features of the tissue of origin [36]. A classical epithelial organoid approach is extracellular matrix-based dome culture. In the original intestinal organoid system, single Lgr5+ stem cells were embedded in Matrigel and maintained with EGF, Noggin, and R-spondin 1 [37]. This format strongly supports self-organization and remains a standard approach for many epithelial organoid systems [37]. One limitation of conventional 3D organoid culture is the restricted access to the apical or luminal compartment [38]. Another limitation is the ill-defined and variable composition of Matrigel [39].

Rotating flasks or spinning bioreactors are used when improved oxygen and nutrient exchange is needed [40]. In the original cerebral organoid workflow, Matrigel droplets were transferred to a spinning bioreactor to enhance nutrient absorption and support further tissue growth [40]. The main advantage of this approach is the improved mass transport for larger tissues [40]. Its main limitation is greater technical complexity. Rotating suspension systems may also introduce shear-related effects [35].

Microwell/AggreWell-type platforms are mainly used to generate size-controlled stem cell aggregates or embryoid bodies before downstream organoid differentiation [41]. Their main advantage is improved uniformity and scalability at the aggregation stage [41]. Their main limitation is that they primarily standardize the starting aggregate, rather than replacing later tissue-specific differentiation and maturation conditions [41].

ODMs are generated by dissociating 3D organoids and seeding the cells onto coated plastic or permeable supports [42]. This format allows separate access to the apical and basolateral sides and supports barrier, permeability, and transport measurements [42]. Its main advantage is experimental accessibility [42]. A key trade-off is reduced 3D architecture compared with intact organoids [38].

OoC/MPS are microengineered culture systems that place cells or tissue constructs in microfluidic environments with defined flow and perfusion [43]. Their main advantage is precise control of exposure, flow, and compartmental coupling [43]. Their main limitation is greater design and operational complexity than conventional static culture [43]. Together, these formats are related but distinct in vitro platforms.

## 3. Scope and Evidence-Mapping Approach

This review provides a structured, mechanism-anchored synthesis of how human organoids (3D and ODM formats) and OoC/MPS have been used to assess biotoxins along the food–water–infection continuum. Our aim is to organize the available primary evidence in a way that is interpretable across platforms, rather than to deliver an exhaustive systematic review or a pooled quantitative estimate of effect.

### 3.1. Toxin Selection and Handling of Gaps

We focus on representative toxins within major public-health biotoxin classes (bacterial enterotoxins, mycotoxins and plant toxins, and algal/cyanobacterial toxins) chosen for (i) well-defined “gatekeeper” mechanisms that advanced in vitro systems can directly interrogate (e.g., vectorial secretion/transport, transporter-dependent uptake, or network electrophysiology), (ii) cross-organ relevance across gut–liver–kidney–brain axes, and (iii) sufficient organoid/ODM/OoC literature to support a structured discussion. Plant toxins are discussed together with mycotoxins in Section 5.2 for organizational purposes. The current organoid/ODM/OoC evidence mapped in this review is limited and is represented mainly by biliatresone in a related hepatobiliary/epithelial context. This scope is intentionally mechanism-anchored rather than comprehensive across all foodborne hazards or toxicants. Viruses are not included as a toxin class in this review because food safety frameworks generally classify them as foodborne pathogens or other infectious/biological hazards, whereas naturally occurring toxins are addressed separately as chemical hazards [44]. Viral infection is discussed only when it modifies toxin-associated outcomes in human-relevant models. Where organoid/OoC evidence is sparse, we explicitly describe the evidence gap rather than extrapolating beyond the available data.

### 3.2. Evidence Emphasis and Species Scope

We prioritize primary studies in which (i) toxin identity and exposure context are clearly defined (rather than generic “mycotoxin” or “algal toxin” labels), and (ii) the biological model is characterized to support mechanistic interpretation (documented polarity/barrier function, transporter competence, secretory response, or electrophysiological maturity as relevant). Although the translational focus of this review is on human systems, we occasionally reference non-human (e.g., porcine, bovine, or murine) organoid studies. Such studies are included only when they validate conserved gatekeeper biology, establish exposure geometry paradigms (apical vs. basolateral; lumenal microinjection; flow configurations), or provide technical benchmarks that are not yet available in human systems. Cell line spheroids and non-human organoids are therefore used here mainly for technical feasibility, comparative mechanism, or route-benchmarking purposes. They should not be interpreted as direct surrogates for human risk assessment. Farm animal organoids are particularly valuable for comparative and veterinary toxicology, but their quantitative effect levels are not assumed to be human-equivalent. Translational interpretation and decision support conclusions in Section 6 and Section 7 prioritize human data whenever available.

### 3.3. Evidence Charting and Quality Lens

Across the studies discussed, we track a consistent set of descriptors that support cross-study interpretability in advanced in vitro systems. These include platform format, exposure geometry and kinetics, biological qualification, dosimetry reporting, primary endpoints, and the specific mechanistic/translational claim. We do not treat model complexity as an inherent marker of evidentiary strength. Instead, interpretability is judged primarily by fit-for-purpose biological qualification, exposure realism/geometry, dosimetry fidelity, and endpoint specificity, consistent with widely used good-practice concepts for in vitro methods (e.g., OECD GIVIMP and GCCP 2.0) [32,33]. For example, a simple but well-qualified polarized 2D/ODM system may provide stronger mechanistic evidence for a toxin claim than a more complex 3D organoid. This is the case when the simpler system has verified pathway competence, controlled apical-versus-basolateral exposure, and quantitative functional readouts, while the more complex organoid lacks documented polarity, delivered-dose information, or pathway-relevant functional endpoints. Added complexity is justified only when it is expected to change interpretation, for example by introducing flow, organ–organ coupling, or subepithelial or vascular compartments that are mechanistically central to the question.

## 4. Cross-Cutting Principles for Interpretable Biotoxin Testing in Organoid-Based Formats and OoC/MPS

**Figure 1:**
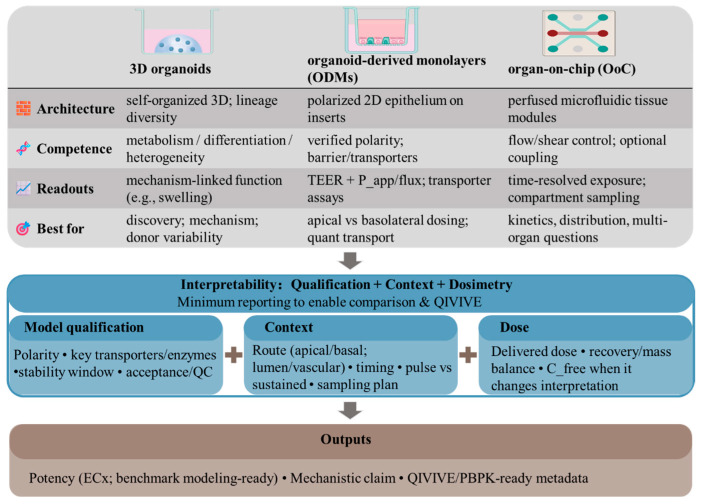
Platform tiers and key determinants for interpretable biotoxin assessment across 3D organoids, ODMs, and perfused OoC/MPS, including a study-at-a-glance reporting minimum for comparability and QIVIVE readiness.

To orient readers to platform selection and the critical determinants of interpretability across tiers, we summarize platform options and a minimal reporting set in Figure 1.

### 4.1. Biological Qualification: Aligning Model Competence with the Toxin Mechanism

Mechanistically interpretable biotoxin studies should explicitly document that the test system expresses the minimal functional competencies required by the toxin’s established mode of action. For enterotoxins, this typically includes polarized ion/water transport capacity (e.g., CFTR- or guanylate cyclase-C (GC-C)-linked secretion); for hepato-/nephrotoxins, it includes relevant uptake/efflux transporters and metabolic competence; and for neurotoxins, it includes stimulus-responsive network activity in addition to viability or morphology. General good-practice frameworks for advanced in vitro systems emphasize this “essential characteristics/fit-for-purpose” approach and its documentation requirements, and can be directly applied to organoids, ODMs, and OoC/MPS used as NAMs [32,33]. In practice, this means documenting the organoid/OoC features that are mechanistically required under assay conditions. These include polarity and barrier integrity for enterotoxin studies, relevant lineage markers together with transporters, enzymes, or receptors for uptake-limited hepato-/nephrotoxin studies, and stimulus-responsive electrophysiology for neurotoxin studies. For OoC/MPS, additional features that affect interpretation should also be documented, including flow, exposure route, and key material/metadata reporting.

### 4.2. Exposure Geometry and Kinetics: Polarity and Time-Varying Dosing as Major Determinants of Interpretation

Because many biotoxins act in a route-dependent manner, exposure geometry (apical/luminal vs. basolateral/vascular) should be treated as a primary experimental design variable rather than a procedural detail. Organoid-derived monolayers on permeable supports provide a practical way to enforce and verify polarity and to obtain quantitative barrier/transport readouts (e.g., TEER and tracer flux) under controlled apical vs. basolateral dosing.

OoC/MPS platforms can further impose perfusion and controlled exposure histories (e.g., sustained low-dose vs. pulse-like dosing). They can also enable organ–organ coupling when systemic distribution is central to the question. Studies should clearly state what causal question the added kinetic control is intended to address [24]. For intestinal epithelial OoC/MPS, studies should also report flow rate, the approximate shear context, and any pulse duration or washout interval used. In representative gut chip designs, epithelial shear is typically low and is often around ~0.02 dyn/cm^2^; published examples span ~0.005–0.04 dyn/cm^2^ depending on channel geometry and flow conditions [45]. For feasibility, long-term luminal perfusion of human intestinal organoids has been demonstrated using dedicated flow chip designs, supporting multi-day exposure paradigms when needed [46]. In the GOFlowChip study, steady-state luminal flow was maintained for as long as 65 h [46]. In conventional closed basal-out organoids, compounds added to the surrounding medium can contact the basolateral-facing exterior without dissociation. However, the apical/luminal compartment remains enclosed. This limits direct comparability with independently addressable apical-versus-basolateral dosing in ODMs or OoC/MPS. Direct apical access generally requires microinjection, polarity reversal, or conversion to monolayer/chip formats [47].

### 4.3. Dosimetry and Material Interactions: Reporting Delivered Exposure, Not Only Nominal Dosing

Quantitative claims about potency, thresholds, or cross-platform differences depend on dosimetry. In organoid/ODM assays as well as in perfused OoC/MPS, nominal dosing can diverge from what the tissue experiences over time. This can result from binding to proteins, extracellular matrix (ECM) domes/hydrogels, or surface coatings, as well as from degradation, adsorption/absorption to plastics or device materials, and incomplete mixing. Therefore, interpretation should be anchored in delivered exposure at the tissue interface and, when relevant, the unbound fraction rather than nominal concentration alone [48,49,50].

At a minimum, studies should state what is known (and unknown) about delivered exposure under their exact assay conditions. Conclusions should be framed accordingly. Conceptually, the goal is not to measure everything in every paper. Rather, it is to avoid unqualified dose–response claims when delivered exposure is likely to differ meaningfully from nominal dose. This issue is often particularly salient in microfluidic systems and polymer-based devices, but is not unique to them [25,51].

### 4.4. Endpoint Harmonization and Benchmarking: A Minimum Comparable Set Plus Toxin-Specific Extensions

Cross-study synthesis requires a small shared endpoint core that is interpretable across platforms, supplemented by toxin-specific mechanistic readouts only when they change the answer. In practice, a comparable core should include 3–5 prespecified functional endpoints. A practical shared menu includes: (i) viability/cell injury (e.g., LDH release or an ATP-based viability assay); (ii) barrier/transport function [TEER plus tracer flux or P_app where a barrier epithelium is present]; (iii) a limited inflammatory panel (e.g., IL-8 with one or two prespecified companion cytokines) when infection or barrier injury is central; and (iv) one organ-level functional readout linked to dominant pathophysiology. Functional endpoints should be prioritized over purely descriptive imaging. Examples include secretion, barrier/transport, differentiated organ function, and electrophysiology. Imaging and molecular markers are best used to support the mechanism and to document model competence and stability. Toxin-specific extensions should be added only when they directly test the claim and are expected to change interpretation. For example, in uptake-limited OTA kidney MPS, transporter perturbation added to a viability core can distinguish uptake-dependent nephrotoxicity from nonspecific cytotoxicity [13]. In Deoxynivalenol (DON) gut chip studies, IL-8 measured alongside TEER revealed a route-dependent inflammatory response that was not captured by barrier readouts alone [52]. Harmonization around a minimal core improves comparability and reduces selective reporting bias, consistent with OECD GIVIMP and GCCP 2.0 principles [32,33].

### 4.5. Translation Readiness: Designing Studies That Can Support Quantitative In Vitro–In Vivo Extrapolation (QIVIVE)

Translation readiness is largely a reporting and study design choice: documenting time-resolved exposure conditions, producing concentration–response information that can be re-used (rather than only significance testing), and clearly separating biological from technical replication. These elements allow later in vitro–in vivo extrapolation (IVIVE) and physiologically based pharmacokinetic (PBPK) and QIVIVE analyses even if modeling is not performed in the original study, and they make results auditable and reproducible [32,33].

Section 5, Section 6 and Section 7 apply these cross-cutting principles to the mapped evidence and then translate them into a minimum, reviewer-auditable implementation standard for future studies.

## 5. Evidence Across Biotoxin Classes

### 5.1. Bacterial Enterotoxins (Cholera Toxin, Heat-Stable Enterotoxin STa, Shiga Toxins)

**Cholera toxin (CT):** Primary human intestinal organoids (enteroids) recapitulate CFTR-dependent secretory pathways relevant to cholera. In cystic fibrosis and non-cystic fibrosis (CF) enteroids, forskolin-induced swelling (FIS) or CT-induced swelling provides a quantitative readout of CFTR-mediated anion/water secretion; multivalent GM1-mimetic CT inhibitors have been ranked in this assay with half maximal inhibitory concentration (IC_50_) values spanning ~15 pM to 9 mM, establishing a mechanism-anchored, organoid-based pharmacology platform [26,53]. Human jejunal enteroids and organoid-derived monolayers from multiple donors have further been used to dissect how glycosphingolipids (GM1) and glycoproteins cooperate as CT receptors. These studies revealed donor-specific variation in glycoconjugate expression, CT binding, cyclic adenosine monophosphate (cAMP) signaling, and intoxication responses [54]. Jejunal crypt-derived porcine intestinal organoids have been differentiated into 2D monolayers on Snapwell inserts that develop increasing transepithelial electrical resistance and show active glucose- and chloride-dependent transport. In Ussing chambers, apical CT exposure induces the expected rise in the short-circuit current, consistent with enhanced CFTR-mediated chloride secretion and validating barrier-level toxin responsiveness in this large-animal organoid model [55]. Basal-out and apical-out porcine intestinal organoids have also been generated and characterized for barrier integrity and permeability (e.g., fluorescein isothiocyanate (FITC)–dextran flux and ethylenediaminetetraacetic acid (EDTA) challenge). This provides a fully 3D platform that could be applied to CT and other secretory enterotoxins, although CT has not yet been systematically profiled in this configuration [56]. More recently, human colonoids have been used as a CT-inducible swelling model to evaluate candidate anti-secretory agents such as the fungal metabolite nornidulin [57]. Nornidulin significantly suppresses forskolin- and CT-induced fluid secretion, demonstrating the utility of organoid systems for inhibitor discovery beyond GM1-based scaffolds. Key study designs, exposure geometries, and decision-informative functional endpoints across toxin classes are summarized in Table 1. Non-human organoid studies are included only when they establish conserved gatekeeper biology or exposure geometry benchmarks that are not yet available in human systems.

**Enterotoxigenic *E. coli* (ETEC) heat-stable enterotoxins (STs):** Murine and human small-intestinal enteroids that express guanylate cyclase-C (GC-C/GUCY2C) have been established as models of ETEC heat-stable enterotoxin-induced secretion. Exposure to synthetic ST peptides (“ST core”) or the therapeutic GC-C agonist linaclotide induces marked cyclic guanosine monophosphate (cGMP) production and fluid accumulation in 3D enteroids. By contrast, *Gucy2c* knockout or pharmacologic CFTR inhibition abrogates these responses, showing that enteroids faithfully recapitulate the canonical GC-C → cGMP → protein kinase G (PKG) → CFTR axis of secretory diarrhea in the near-native epithelium [27].

Patient-derived pediatric sigmoid colonoids have extended these findings to the human colon. In colonoids from children with disorders of gut–brain interaction, the GC-C agonist linaclotide increases cGMP levels and induces colonoid swelling. This confirms that GC-C-driven secretory responses described in small-intestinal enteroids also operate in human colonic organoids. Importantly, microbiota-derived butyrate significantly dampens linaclotide-induced cGMP elevation and swelling. This suggests that luminal metabolites can tune GC-C pathway activity and drug responsiveness in a donor-specific manner [58].

Complementary work in intestinal epithelial monolayer models has shown that prolonged intoxication with ETEC heat-stable toxins leads to substantial extracellular cGMP accumulation. This, in turn, drives epithelial IL-33 induction and alters downstream innate and adaptive intestinal immune responses, thereby mechanistically linking the ST–GC-C axis to mucosal cytokine and alarmin signaling [59]. Because this IL-33 work was performed mainly in T84 monolayers and mouse models rather than organoids, we treat it here as a downstream mechanistic extension rather than as core organoid evidence [59]. The organoid data define the canonical ST → GC-C → cGMP → PKG → CFTR secretory branch of diarrheal physiology [27,58]. By contrast, the IL-33 findings suggest that prolonged ST exposure may additionally engage extracellular cGMP-linked cytokine/alarmin signaling beyond the acute secretory response [59].

Although ST-specific OoC studies are still scarce, the detailed GC-C pharmacology mapped in enteroids and patient-derived colonoids provides a clear blueprint for intestine-on-chip platforms seeded with organoid-derived epithelia. This is exemplified by “organoids-on-a-chip” and gut-organoid flow chip systems in which perfusion, shear stress and luminal versus basolateral exposure can be precisely controlled to study secretion, transport and host–pathogen interactions under more physiological conditions [46,60].

**Shiga toxins (Stx) from enterohemorrhagic *Escherichia coli* (EHEC)/Shiga toxin-producing *E. coli* (STEC):** Induced human intestinal organoids (iHIOs) generated from pluripotent stem cells have been used to model infection by commensal versus Shiga toxin-producing *E. coli*. In this system, commensal strains replicate within the lumen without disrupting the epithelium. By contrast, O157:H7 infection rapidly disturbs F-actin-rich epithelial structures, compromises barrier integrity, increases reactive oxygen species, and induces IL-8 and other inflammatory responses. The addition of human neutrophils to infected iHIOs further recapitulates neutrophil recruitment and amplifies epithelial damage, thereby capturing key features of early EHEC disease in a primary human 3D system [61].

**Table 1:** Representative organoid-based formats, ODMs, and fit-for-purpose MPS studies for biotoxin assessment.

Toxin Class	Toxin	Study Focus	Source	Model/Culture Platform	Exposure Setup	Primary Endpoints	Key Finding	Ref
Bacterial enterotoxins	CT	Inhibitor screen	Human primary	Intestinal organoids; 3D ECM-embedded organoids; static	CT 10 ng/mL; 4 h	Swelling; IC50	Organoids enabled quantitative ranking of CT inhibitors	[26]
Bacterial enterotoxins	CT	Receptor usage/donor variation	Human primary	Jejunal enteroids; 3D expansion followed by ODMs; static	Apical CT challenge; 5 h intoxication readout in monolayers	CT binding; intoxication/Isc	Donor glycoconjugates altered CT binding and intoxication	[54]
Bacterial enterotoxins	CT	Secretory transport benchmark	Pig primary	ODMs on Snapwell inserts; Ussing chamber; static	Mucosal CTX 7.5 μg/mL; 80 min	Isc; Rt	CT increased secretory current and reduced epithelial resistance	[55]
Bacterial enterotoxins	CT	Anti-secretory compound	Human primary	Human colonoids; 3D ECM-embedded organoids; static	CT 2 μg/mL ± nornidulin 40 μM; 240 min	Swelling	Nornidulin suppressed CT-induced fluid secretion	[57]
Bacterial enterotoxins	STa	GC-C signaling; linaclotide comparator	Human + mouse primary	Enteroids; 3D ECM-embedded organoids; static	STa or linaclotide; cGMP assay 10 μM for 24 h; swelling assay 2 h	cGMP; swelling; CFTR dependence	Enteroids reproduced the GUCY2C–cGMP–PKG–CFTR axis	[27]
Bacterial enterotoxins	Shiga toxin-producing *E. coli* O157:H7 (infection model)	Luminal infection ± neutrophils	Human PSC-derived	iHIOs; 3D; luminal microinjection; static	~10^3^ bacteria; 4 h primary infection readouts	Epithelial injury; ROS; IL-8; neutrophil recruitment	O157:H7 disrupted epithelium and promoted neutrophil-associated injury	[61]
Bacterial enterotoxins	Shiga toxins (Stx1, Stx2a)	Route effects	Human PSC-derived	HIOs (3D; luminal microinjection or basolateral medium exposure) plus enteroid monolayers/transwells; static	Route comparison assays used 30 ng toxin; barrier kinetics assessed up to 72 h; several cell death/mesenchymal assays used 27 h incubations	Cell death; permeability; transcytosis	Exposure route altered tissue injury and apical-to-basolateral toxin movement	[62]
Bacterial enterotoxins	Shiga toxin-producing *E. coli* O157:H7 (infection model)	Colon chip pathogenesis under flow	Human primary epithelium + endothelium	Colon chip with primary human colon epithelium and endothelium; perfused microfluidic device	24 h microbiome–metabolite pre-exposure; 1.7 × 10^5^ EHEC; 3 h static attachment +24 h perfusion	Lesion area; epithelial detachment; cytokines/chemokines	Human microbiome metabolites enhanced EHEC pathogenesis	[63]
Mycotoxins/plant toxins	AFB1	Bioactivation/DNA adducts	Human primary tissue-derived	Gastric, liver, kidney, and colon organoids; 3D ECM-embedded organoids; static	48 h; tissue-specific viability-calibrated dosing	DNA adducts; γ-H2AX/p53/p21; CYP expression	Human organoids bioactivated AFB1; liver showed the strongest adduct/DNA-damage response	[64]
Mycotoxins/plant toxins	Biliatresone	Cholangiocyte injury/cilia	Human infant primary	Human liver organoids; 3D ECM-embedded organoids plus cholangiocyte monolayer on 6-channel ibidi flow chip	2 μg/mL; days 0–5 in organoids; shear flow assay at 1 dyne/cm^2^ on chip	HNF4A; MDR1-R123; FITC-dextran; Ca^2+^ flow response; cilia	Biliatresone induced BA-like defects in polarity, barrier function, cholangiocyte differentiation, and mechanosensing	[29]
Mycotoxins/plant toxins	DON	Route comparison	Mouse primary	Enteroids; 3D ECM-embedded organoids; luminal microinjection vs. basolateral medium exposure; static	Final DON 1 μM; luminal route used 28.3 μM microinjection solution to achieve 1 μM final luminal exposure; stem cell readouts 24 h; permeability tracked to 96 h; junction marker timing reported as prolonged exposure, with text/figure inconsistency (48–72 h)	FITC–dextran influx; junction proteins; Lgr5/EdU	Basolateral DON caused stronger injury than luminal DON	[65]
Mycotoxins/plant toxins	DON	Apical-out bovine organoids; LCS rescue	Bovine primary	Apical-out organoids; suspension culture without Matrigel; static	DON 25 μM for 6 h; ± LCS co-treatment	Viability; barrier integrity; LGR5/Ki67/Mucin2/Villin2/E-cadherin	LCS partially rescued DON toxicity and barrier disruption	[66]
Mycotoxins/plant toxins	OTA	Proximal tubule MPS nephrotoxicity/transport	Human primary	Primary human PTEC kidney MPS; perfused microfluidic tubule; vascularized dual-channel MPS used for transport studies	OTA 0–100 μM; 48–168 h across donors; mechanistic studies at OTA 10 μM with ABT 1 mM or NBDHEX 3 μM;	Viability; RNA-seq; transport/perturbation readouts	OTA toxicity was time-dependent and modulated by transport and metabolism	[13]
Algal/cyanobacterial toxins	DA	Neural MPS benchmark	Human iPSC-derived	BrainSpheres on HD-MEA; multiple spheroids per well; ROI-based 3D neural MPS readout	13-day exposure; 0.1, 0.3, 1, 10, 30 μM	Network firing/burst/synchrony	DA served as a functional neurotoxicity benchmark	[30]
Algal/cyanobacterial toxins	STX	Developmental neurotoxicity synergy with ZIKV	Human iPSC-derived	Brain organoids; brief Matrigel coating early, then ultra-low-attachment/agitation culture; static	50-day-old organoids; ZIKV MOI 0.5 for 2 h, then STX 12 μg/L for 13 days	TUNEL; progenitor-zone injury; viral replication	STX exacerbated ZIKV-induced neural injury	[31]

**Abbreviations**: 3D, three-dimensional; ABT, 1-aminobenzotriazole; AFB1, aflatoxin B1; Ca^2+^, calcium; CFTR, cystic fibrosis transmembrane conductance regulator; cGMP, cyclic guanosine monophosphate; CT, cholera toxin; DA, domoic acid; DON, deoxynivalenol; ECM, extracellular matrix; EdU, 5-ethynyl-2′-deoxyuridine; EHEC, enterohemorrhagic *Escherichia coli*; FITC–dextran, fluorescein isothiocyanate–dextran; GC-C, guanylate cyclase C; HD-MEA, high-density microelectrode array; HNF4A, hepatocyte nuclear factor 4 alpha; IC50, half-maximal inhibitory concentration; iHIOs, induced human intestinal organoids; IL-8, interleukin-8; iPSC, induced pluripotent stem cell; Isc, short-circuit current; LCS, Lactobacillus plantarum culture supernatant; LGR5, leucine-rich repeat-containing G protein-coupled receptor 5; MDR1, multidrug resistance protein 1; MOI, multiplicity of infection; MPS, microphysiological system; NBDHEX, 6-(7-nitro-2,1,3-benzoxadiazol-4-ylthio)hexanol; ODM, organoid-derived monolayer; OTA, ochratoxin A; RNA-seq, RNA sequencing; ROS, reactive oxygen species; Rt, transepithelial resistance; STa, heat-stable enterotoxin A; STX, saxitoxin; Stx, Shiga toxin; TUNEL, terminal deoxynucleotidyl transferase dUTP nick end labeling; ZIKV, Zika virus; γ-H2AX, phosphorylated histone H2AX; p-p53, phosphorylated p53.

Using genetically matched human intestinal organoids and enteroids of differing complexity, Pradhan and colleagues exposed tissues to purified Stx1a and Stx2a from either the luminal or basolateral side. They showed that both toxins induce necrosis and apoptotic death in epithelial and surrounding mesenchymal cells, accompanied by dose- and time-dependent increases in epithelial permeability. In addition, Stx2a microinjection into HIOs in vitro and in transplanted HIOs in vivo produced epithelial damage, hemorrhage, and paradoxical up-regulation of several tight-junction and structural proteins. These findings highlight complex barrier responses and mesenchymal–epithelial crosstalk in Stx-mediated injury [62].

Complementary polarized in vitro models using primary human colonic cells have quantified Stx translocation and barrier disruption at the tissue scale. One study compared a simple epithelial monolayer with a three-layer model containing colonic epithelium, myofibroblasts, and microvascular endothelium. Only ~0.01% of apically applied Stx1a or Stx2a crossed the epithelial-only monolayer, whereas up to ~0.09% traversed the three-layer construct. In both models, Stx2a translocated about 3–4-fold more efficiently than Stx1a, and the more biomimetic three-layer model exhibited substantially greater Stx passage. These results underscore the importance of subepithelial compartments in governing Stx movement across the intestinal wall [67].

Microphysiological OoC systems further add controlled flow and shear that reshape host–pathogen/toxin dynamics relative to static cultures. In a human colon chip lined with primary human colonic epithelium derived from patient tissue and a parallel vascular channel of human intestinal microvascular endothelial cells, EHEC infection under flow reproduces key features of colonic injury, including epithelial barrier loss and inflammatory cytokine release. The model also revealed that microbiome-derived metabolites from humans, compared with those from mice, potentiate epithelial damage by up-regulating EHEC flagellin and motility/virulence programs. This highlights important non-Stx contributors to pathogenesis in a human microphysiological context [63].

A fluidically coupled gut → kidney axis-on-chip model using Caco-2 cells in an intestinal module and HKC-8 proximal tubule cells in a kidney module has been used to link O157 infection, Stx2 release, and downstream renal injury. In this model, purified Stx2 applied to the gut channel reduced HKC-8 viability and compromised barrier properties in the kidney module, even though pharmacokinetic modeling predicted that only ~0.12% of the initial gut-side Stx2 concentration would reach the kidney compartment after 72 h. When the gut module was infected with O157 and then treated with antibiotics, ciprofloxacin versus gentamicin produced distinct Stx2 release profiles and differences in kidney module toxicity. These findings illustrate how antibiotic choice can modulate systemic Stx exposure and risk of hemolytic uremic syndrome [28].

**Knowledge gaps and outlook (enterotoxins):** Taken together, CT, STa and Stx have been interrogated across a spectrum of organoid-based platforms with endpoints including swelling, cGMP, short-circuit current, TEER, tracer flux and histologic injury, but most studies are still single-toxin and single-organ in scope. Systematic, donor-diverse potency panels that apply harmonized readouts (e.g., FIS and TEER/flux) across CT, STa and Stx in human intestinal organoids and organoid-derived monolayers are still lacking. The same is true for intestine-on-chip and coupled gut–kidney systems that implement realistic luminal dosing (meal-like pulses, mucus and shear). Such platforms would be critical for comparing toxin variants and for testing prophylactic or therapeutic interventions in a more translational framework. Priority next steps include extending antibiotic-modulated Stx-release studies from current gut–kidney chip systems to pediatric or donor-diverse human intestinal organoid/ODM models. Another priority is to establish donor-diverse colonoid panels to quantify microbiota-tuned GC-C responses [28,58]. To strengthen translational relevance, these studies should predefine clinically anchored outputs. These outputs include stool pathogen burden in ETEC diarrhea and, where feasible, fecal toxin measurements in Stx-linked disease [68,69].

### 5.2. Mycotoxins and Plant Toxins

**Aflatoxin B1 (AFB1):** Human tissue organoids derived from the stomach, liver, kidney and colon can metabolically activate AFB1 and form measurable AFB1 DNA adducts [64]. Adduct burdens are higher in liver organoids, the in vivo target tissue, than in gastric organoids, a non-target tissue. This supports the use of these organoid systems for human-relevant bioactivation and tissue-specific susceptibility comparisons [64]. In the same system, AFB1 exposure elicits cytotoxicity and activates DNA-damage signaling, including γ-H2AX, phospho-p53 and p21 [64]. It also induces key xenobiotic-metabolizing enzymes, including CYP1A1, CYP1A2, CYP3A4 and NQO1 [64]. Together, these findings support the use of human liver organoids as high-relevance models for AFB1 metabolic activation and genotoxicity [64]. In the cited study, 48 h viability assays yielded gastric organoid IC_50_ values of 22 μM and 126 μM, whereas liver organoids did not reach an IC_50_ of up to 150 μM [64]. These concentrations are therefore discussed here as nominal exposure conditions. Multi-organ MPS that fluidically couple liver spheroids with downstream tissues can enable tests of how hepatic biotransformation reshapes downstream responses to pro-toxins such as AFB1 [70,71].

**Ochratoxin A (OTA):** Human kidney microphysiological systems (MPS) seeded with primary human proximal tubule epithelial cells have been used to model OTA-induced nephrotoxicity [13]. In a 3D kidney proximal tubule MPS, OTA LC_50_ values of 0.38–1.21 μM were reported. The authors noted that these values were in agreement with clinically relevant toxic OTA concentrations in human urine [13]. Co-treatment with the pan-P450 inhibitor 1-aminobenzotriazole decreased OTA-induced toxicity, whereas the glutathione S-transferase (GST) inhibitor NBDHEX increased toxicity. This suggests that cytochrome P450-mediated oxidative activation and GST-mediated conjugation both shape OTA bioactivation and detoxification in this system [13]. These LC_50_ values are discussed here as nominal exposure conditions. Measured free fractions were not reported [13].

In the same work and in a subsequent kidney organoid/MPS review, these primary proximal tubule epithelial cell (PTEC)-based MPS, including a vascularized proximal tubule MPS (VP-MPS), have been highlighted as transporter-competent nephrotoxicity models [13,72]. In the VP-MPS, basolateral OTA uptake was mediated primarily by organic anion transporters OAT1/3. This conclusion was supported by transport experiments with the OAT inhibitor probenecid and by basolateral OAT1 expression. Together, these findings show that such platforms can dissect transporter-dependent OTA disposition under flow [13,72].

In parallel, a simple microfluidic “SpheroFlow Device” hosting 3D human neuroblastoma SH-SY5Y spheroids has been used to examine acute OTA and patulin toxicity under continuous perfusion [73]. This study shows that dynamic on-chip exposure of 3D cultures to mycotoxins is technically feasible. It also provides reproducible concentration–response data for viability endpoints [73].

**Deoxynivalenol (DON):** Three-dimensional mouse small-intestinal organoids (enteroids) have been used to compare luminal versus basolateral DON exposure. Microinjection of DON into the lumen versus addition to the basolateral medium showed that basolateral exposure at 1 µM more strongly disrupts barrier function. Readouts included FITC–dextran influx and altered tight-junction proteins, including E-cadherin, zonula occludens-1 (ZO-1), claudin-2, and occludin. Basolateral exposure also more strongly suppressed Lgr5^+^ stem cells and proliferating cells. These findings establish route-dependent DON effects in a murine system, but their quantitative effect levels should not be interpreted as human-equivalent thresholds [65].

Apical-out bovine intestinal organoids have also been established as an alternative model for evaluating DON toxicity and probiotic detoxification. In this system, luminal DON exposure reduces organoid viability and barrier integrity. Co-treatment with probiotic *Lactobacillus* culture supernatant partially restores these endpoints. These bovine data are therefore most informative as comparative or farm animal evidence rather than as direct human risk estimates [66,74].

A 3D human gut-on-a-chip model with flow (OrganoPlate 3-lane) further demonstrated dose- and route-dependent DON effects. Continuous TEER and barrier integrity assays showed that DON impaired barrier function at higher nominal concentrations under flow than in static models. The barrier was also more sensitive to basolateral than apical exposure. By contrast, IL-8 secretion was induced mainly by apical DON and was not captured by TEER changes. This finding highlights how shear and exposure geometry reshape DON responses [52]. This caution is important because DON metabolism is species-dependent. Both glucuronidation and intestinal microbiota-mediated de-acetylation differ across species, which can alter effective intestinal exposure and barrier responses [75,76].

Taken together, current DON studies using intestinal organoids and gut-on-a-chip platforms remain relatively few and heterogeneous in exposure route, dosing schemes and readouts. More standardized apical dosing protocols and harmonized barrier (TEER/permeability, tracer flux) and cytokine panels across donors and platforms would facilitate robust comparison and integration of organoid- and chip-based data [52,65,66,74]. Formal fitted IC_50_/half-maximal effective concentration (EC_50_) values were not reported in the cited DON studies. In the human gut chip study, DON was applied at nominal concentrations of 3–300 μM [52]. Cross-study interpretation is therefore anchored mainly to barrier and cytokine readouts rather than to directly comparable potency metrics.

**Biliatresone (plant-derived isoflavonoid):** In immortalized and primary murine cholangiocyte spheroid models, the plant isoflavonoid biliatresone causes rapid biliary damage [77]. It disrupts spheroid morphology, closes lumens, mislocalizes the apical tight-junction marker ZO-1, increases leakage of luminal rhodamine 123, and leads to monolayer disruption and loss of apical F-actin [77]. Mechanistic follow-up studies further show that biliatresone-induced depletion of intracellular glutathione (GSH) activates a linear signaling cascade. Increased RhoU/Wrch1 and Notch effector Hey2 drive down-regulation of SOX17, linking redox stress to Wnt/Notch signaling, cholangiocyte injury, and bile duct obstruction in a toxic model of biliary atresia [77,78].

In primary human liver organoids derived from normal infant liver tissue, biliatresone exposure reduces organoid expansion and skews differentiation [29]. It reduces expression of the cholangiocyte marker CK19 and increases HNF4A^+^ hepatocyte-like cells. It also disrupts tight-junction and cytoskeletal organization, impairs multidrug resistance protein 1 (MDR1)-mediated luminal transport and increases epithelial permeability to FITC–dextran. Together, these changes produce morphological and functional abnormalities that resemble previously reported biliary atresia (BA) organoids [29]. In the same human organoid study, biliatresone also reduces the proportion of ciliated cholangiocytes and abolishes primary cilium-dependent mechanosensory calcium responses when organoid-derived cholangiocyte monolayers on a microfluidic chip were exposed to shear flow [29]. The cited human liver organoid study used a single nominal biliatresone concentration of 2 μg/mL [29]. Neither the cited human nor the cited murine biliatresone studies reported fitted IC_50_/EC_50_ values [29,77,78]. Cross-study interpretation is therefore based mainly on morphology, permeability, transport, and ciliary function endpoints rather than on directly comparable potency metrics.

Unless otherwise stated, concentrations in this section are discussed as nominal exposure conditions. Measured free fractions were not reported in the cited AFB1, DON, or biliatresone studies. For OTA, the cited kidney MPS study reported LC_50_ values and mechanistic transport/metabolism data, but not measured free fractions.

**Knowledge gaps and outlook (mycotoxins/plant toxins):** Across AFB1, OTA, DON and biliatresone, current organoid and microphysiological system studies remain relatively sparse and heterogeneous. Exposure designs are often short-term, single-compound, and based on nominal concentrations. Reporting of exposure metrics is also inconsistent. These features make cross-platform comparison and quantitative in vitro-to-in vivo extrapolation (QIVIVE) difficult. To move toward risk assessment-grade applications, future work should systematically quantify and report free (unbound) concentrations, recovery/mass balances across medium, cells, device materials, and other delivered-exposure metrics under the exact assay conditions used. This is especially important for highly protein-bound mycotoxins such as OTA, so that internal exposures can be linked to physiologically based pharmacokinetic (PBPK) models. For AFB1, DON, and biliatresone, the cited organoid studies did not report measured free fractions. For DON and biliatresone, formal fitted IC_50_/EC_50_ values were also not reported. Actionable next steps are threefold. First, chronic binary or ternary mixture designs should be anchored to documented co-occurrence patterns in food and feed, rather than to unspecified co-exposure. [79]. Second, QIVIVE-ready endpoint packages should pair functional readouts with mechanistically anchored exposure metrics. For AFB1, this includes DNA adduct burden together with the delivered dose. For OTA, this includes transporter-qualified uptake dependence together with recovery or mass balance data. For DON, this includes route-specific TEER/flux together with IL-8 under defined exposure conditions. Third, these studies should be implemented under the good-practice and reproducibility frameworks already cited in this review, including OECD GIVIMP, GCCP 2.0, and the MPS reporting/reproducibility frameworks discussed in Section 6 [32,33,80,81,82]. In addition, most existing liver, kidney and gut organoid/MPS studies still use acute, single-toxin exposures, whereas human dietary exposure is typically chronic and involves co-occurring contaminants. There is therefore a clear need for chronic low-dose and mixture protocols. These should combine mycotoxins with relevant co-contaminants or metabolic modifiers, such as CYP3A4/1A2 modulators for AFB1 and OAT inhibitors or competitors for OTA. For prototypical genotoxic carcinogens such as AFB1, longitudinal liver organoid and liver-on-chip studies are needed. These studies should track DNA adduct formation and repair together with functional phenotypes (e.g., proliferation, senescence, transformation markers) and integrate these data into established PBPK–QIVIVE workflows. This would provide a concrete bridge between organoid-scale mechanisms and quantitative cancer-risk estimates [83,84].

### 5.3. Algal/Cyanobacterial (Marine and Freshwater) Toxins

**Saxitoxin (STX):** Human brain organoids infected with Zika virus (ZIKV) have been used to model combined exposure to the cyanobacterial neurotoxin saxitoxin (STX). In 50-day brain organoids, chronic exposure to 12 μg/L STX (12 ng/mL) for 13 days caused little additional neural cell death. However, co-exposure with ZIKV approximately doubled terminal deoxynucleotidyl transferase dUTP nick end labeling (TUNEL)-positive cell death in progenitor zones compared with ZIKV alone. This demonstrates toxin–virus synergy in a human developmental neurotoxicity model [31]. The authors selected this concentration because STX had been reported in untreated water sources during drought conditions in northeastern Brazil. For context, the same study cites STX levels of approximately 0.003–0.766 μg/L in regional reservoirs, a Brazilian drinking water guideline value of 3 μg/L, and 15 ng/L in the mouse drinking water experiment. In the same study, pregnant immunocompetent mice given STX-contaminated drinking water before and during gestation and infected with ZIKV produced offspring with more severe brain malformations than mice exposed to ZIKV alone. These included reduced cortical thickness and enlarged ventricles. This supports the in vivo relevance of the organoid findings [31].

**Domoic acid (DA):** A 3D human iPSC-derived brain microphysiological system (“BrainSpheres”) recorded on a high-density microelectrode array has been used for functional neurotoxicity screening. In this system, domoic acid was used as a positive control excitotoxin. It produced concentration-dependent alterations in network activity metrics, including mean firing rate, burst structure and synchronicity. This establishes an objective functional readout for excitatory neurotoxicants in human 3D neural tissue [30].

**Microcystin-LR (MC-LR):** An advanced 3D human liver model (Hepoid-HepaRG) based on collagen-embedded HepaRG multicellular spheroids shows high expression of OATP1B1 and OATP1B3 and marked sensitivity to MC-LR. In 14-day-old Hepoid-HepaRG cultures, 48 h exposure to ≥10 nM MC-LR caused cytotoxicity. The reported effect concentration producing a 20% response (EC_20_) was approximately 26 nM. MC-LR also impaired albumin secretion, reduced cytochrome P450 1A/1B enzyme activities, and down-regulated hepatocyte differentiation markers including CYP1A2, ALB, HNF4A and several hepatobiliary transporters. These findings support the use of this model as a human-relevant transporter-competent platform for cyanotoxin hepatotoxicity [85].

Scaffold-free spheroids generated from adult human liver stem cells (HL1-hT1) also showed high sensitivity to cyanotoxins. Prolonged exposure to microcystin-LR or cylindrospermopsin at sub-micromolar concentrations (≥0.1 µM) reduced spheroid growth and viability. For MC-LR, the reported 96 h EC_50_ was approximately 0.04 µM. No-observed-effect concentration was below 0.01 µM. Surface blebbing and partial disintegration were also observed. These findings indicate that 3D adult liver stem cell spheroids can detect MC-LR hepatotoxicity at environmentally relevant levels [86].

Together, these 3D liver models indicate that human liver spheroids and organoid-like constructs that preserve OATP1B1/1B3 function are well suited for future organoid- and liver-chip-based studies of low-dose MC-LR. They may also be useful for study in mixtures with other cyanotoxins and interactions with co-medications. A key advantage is that they can incorporate transporter-dependent uptake into toxicodynamic readouts [85,86].

Mechanistic work in human liver systems also shows that MC-LR hepatotoxicity depends on active uptake through OATP1B1/1B3 rather than passive diffusion. The Hepoid-HepaRG spheroid system therefore provides a relevant platform for testing how the pharmacological inhibition or altered function of these transporters change MC-LR uptake and toxicity. This logic can be extended directly to liver-on-chip formats under flow [85].

**Cylindrospermopsin (CYN):** Although the in vitro toxicology literature on CYN is still dominated by 2D cell systems, several human 3D hepatic studies have now been reported. These studies are mainly based on static spheroid models, including scaffold-free HL1-hT1 adult liver stem cell spheroids and HepG2 spheroids [86,87,88]. Based on the currently available literature, CYN does not yet have a broadly adopted liver organoid or liver-chip benchmark. Unlike MC-LR, for which hepatocellular uptake has a clearer mechanistic anchor [89], CYN still lacks a similarly established qualification framework in human 3D hepatic models. CYN is a highly water-soluble zwitterionic toxin [90]. Its toxicological profile includes protein synthesis inhibition, oxidative stress-associated injury, and genotoxic responses [91,92], while the contribution of metabolism remains unresolved across systems: CYP inhibition reduced cytotoxicity or genotoxicity in primary hepatocyte models, whereas no phase I metabolites were detected in HepaRG cells or liver fractions by LC-HRMS [91,92,93]. Fit-for-purpose 3D hepatic studies of CYN should therefore prioritize stable hepatic function, delivered exposure, and mechanism-aligned endpoints beyond bulk viability.

**Knowledge gaps and outlook (algal/cyanobacterial toxins):** For algal and cyanobacterial toxins, near-term priorities include three areas. First, establishing standardized multi-electrode array benchmarks in brain organoids and 3D neural MPS. For example, domoic acid and saxitoxin concentration–response curves on network firing and synchrony would complement purely morphological readouts. Second, designing liver-chip studies that explicitly quantify MC-LR uptake and efflux via OATP1B1/1B3 under flow. These studies should also determine how transporter genotype or pharmacologic inhibition changes tissue dose and toxicity in 3D human liver tissue. Such data could then feed into PBPK/QIVIVE frameworks for internal-dose extrapolation. Third, for CYN, established human 3D hepatic models should be extended under well-documented assay conditions rather than treated as intrinsically unsuitable. This includes confirming stable hepatic function, reporting delivered exposure, and incorporating mechanism-aligned endpoints for protein synthesis inhibition, oxidative stress, genotoxicity, and hepatic functional impairment. Liver-chip or coupled liver–kidney formats may be especially informative when perfusion, exposure kinetics, or inter-organ disposition are expected to change interpretation.

## 6. From Mapped Evidence to Decision Support: Making Organoid/ODM and OoC/MPS Biotoxin Studies Comparable and Translatable

**Figure 2:**
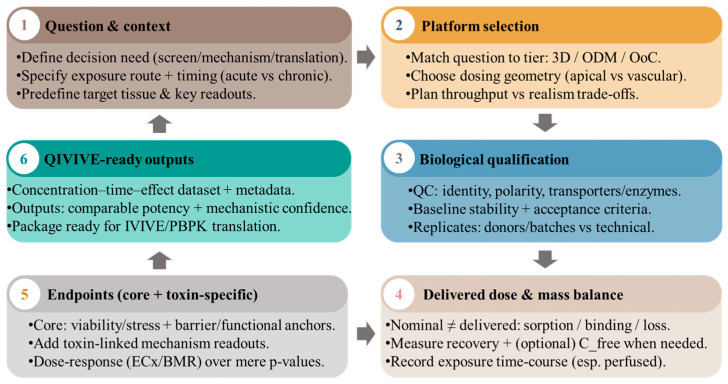
Translational roadmap from question framing to QIVIVE-ready outputs for organoid/ODM and OoC/MPS biotoxin studies.

Section 3 summarizes the guiding principles (what matters and why). Here, we translate them into a minimum implementation standard that reviewers can verify in the Methods and Results. Table 2 provides a concise checklist of the reporting elements and acceptance criteria that, if present, make organoid/ODM and OoC/MPS biotoxin studies comparable and QIVIVE-ready. Figure 2 provides a stepwise translational roadmap from question framing and platform choice to QIVIVE-ready outputs, including when escalation to perfused OoC/MPS is expected to change interpretation.

### 6.1. A Shared Interpretability Logic Across Toxin Classes: Critical Determinants + Functional Endpoints

Across toxin classes, the key decision support question is twofold. First, does the model express the critical determinants of internal dose and susceptibility under the assay conditions? Second, are functional endpoints quantified that map to dominant pathophysiology? When this logic is explicit, platform choice becomes a fit-for-purpose engineering decision rather than an implicit “complexity-equals-relevance” claim. This improves cross-study comparability and reviewer confidence [32,33].

### 6.2. Exposure Truth That Survives Peer Review: Delivered Concentration, Mass Balance, and When Free Concentration Matters

Minimum dosimetry reporting that survives peer review (Table 2) should include: (i) dosing matrix and binding modifiers (e.g., serum/protein, mucus, ECM, additives); (ii) exposure geometry and time profile (apical vs. basolateral; flow rate; pulse vs. continuous; exposure duration); (iii) the concentration metric used for interpretation (nominal, measured bulk, measured inlet/outlet, or estimated/measured tissue-surface exposure); (iv) whether recovery/mass balance across compartments and device was assessed; and (v) relevant material and matrix interactions across the assay format, including plastics, ECM/culture matrices, surface coatings, and, for microfluidic devices, material choice and any mitigation of compound loss [49,50]. When measurement is infeasible, the manuscript should state this explicitly and bound conclusions as nominal dose-anchored rather than implying delivered-dose precision [32,33].

When free concentration (C_free) is likely to change interpretation, the study should address it explicitly. This is particularly important for highly protein-bound toxins and for systems with substantial nonspecific binding. Studies should either report measured or estimated C_free, with the method and compartment clearly specified, or clearly justify interpretation based on total concentrations. C_free is not a universal requirement. It is needed when it changes the mechanistic conclusion or materially improves PBPK/QIVIVE linkage [32,33].

For toxins whose activity depends on biotransformation, the parent compound alone may not define the biologically effective dose. Metabolite contributions may differ across cell types, metabolic competence, transport properties, and culture platforms. As a result, similar nominal exposures can produce different experimental readouts. This is well illustrated by AFB1, which is metabolically activated in human organoids and forms measurable DNA adducts, and by OTA, for which transport and metabolism both influence toxicity in kidney MPS [64]. Where feasible, studies should determine whether major metabolites, conjugates, or adduct-forming species are generated under the assay conditions. They should also report where these species are measured, for example in bulk medium or tissue lysate. Informative designs can combine chemical analysis of parent and metabolite species with the perturbation of metabolism or transport. When standards are available, matched testing of authentic metabolites can provide further support [13].

### 6.3. Fit-for-Purpose Biological Qualification and a Minimal Comparable Endpoint Set

For toxin-specific claims, biological qualification should be proportional to the mechanism invoked. Enterotoxin studies should document polarity and pathway competence. They should also pair secretion readouts with pathway-confirming second messengers. Nephrotoxin studies that invoke basolateral uptake should document transporter competence under assay conditions. Hepatotoxin studies for uptake-limited toxins should verify relevant uptake transporters and confirm the maintenance of differentiated function during the exposure window. Neurotoxin studies should not rely only on morphology or viability. They should include functional electrophysiology when network activity is the mechanistic anchor.

To make evidence comparable across platforms, Table 2 defines a minimal comparable functional endpoint core. Toxin-specific extensions should be added only when they directly test the claim. A practical core set includes: (a) barrier/transport: TEER plus tracer flux (P_app or equivalent); junctional and cytoskeletal markers should be used as mechanistic support; (b) secretagogues/enterotoxins: swelling or electrophysiology, paired with pathway-confirming second messengers (cAMP or cGMP) and appropriate controls; (c) liver/kidney toxins: viability interpreted alongside differentiated function and transporter/enzyme qualification when invoked; and (d) neurotoxins: MEA network metrics paired with viability/developmental markers [80].

QIVIVE-ready reporting is primarily about reusable quantitative outputs (Table 2). These include: (i) concentration–response information with effect levels reported as effect concentration producing an x% response (ECx) or benchmark responses when feasible; (ii) clear definitions of biological replicates (donors/batches) versus technical replicates; (iii) time-resolved exposure metadata (geometry, duration, sampling points, and the concentration metric used); and (iv) sufficient method details to enable later IVIVE/PBPK modeling without re-running foundational dosimetry and concentration–response work.

### 6.4. Designing Now for Translation Later: Building QIVIVE Readiness into Study Design

Even when a study is primarily mechanistic, it can be structured to remain translation-ready. This requires three things. First, studies should document time-resolved exposure, including nominal and delivered concentrations. Second, they should report concentration–response metrics that support EC_x or benchmark responses, rather than relying only on significance testing. Third, they should clearly distinguish biological replicates, such as donors or batches, from technical replicates. These practices facilitate later IVIVE/PBPK workflows that connect in vitro effect levels to human exposure contexts [83]. Importantly, “QIVIVE-ready” does not mean that every paper must perform PBPK modeling. It means that experimental reporting is sufficiently complete that others can perform it later without re-running foundational dosimetry and concentration–response work.

When to escalate from organoid/ODM to OoC/MPS: a fit-for-purpose heuristic and what to report when you do. OoC/MPS should not be viewed as an aesthetic upgrade or as inherently superior to organoid/ODM. Instead, platform choice should be guided by whether engineered microenvironmental features are expected to influence biological interpretation. Microscale culture can itself alter cell behavior by changing volume-to-cell ratios, endogenous signal accumulation or dilution, and the balance between diffusion and convection [94,95]. Microfluidic devices can further reproduce organ-relevant physical cues. These include perfusion, shear stress, oxygen control, compartmentation, and, in some systems, mechanical deformation [48,96]. OoC/MPS is therefore particularly valuable when exposure kinetics are central to the hypothesis. It is also valuable when basolateral delivery or systemic distribution governs tissue dose, when transporter-dependent uptake or efflux under flow is mechanistically important, or when delayed downstream effects in coupled tissues are the primary endpoint. When OoC is used mainly to enforce exposure geometry or enable multi-day luminal exposure paradigms, this should be stated explicitly and treated as the controlled variable. The reason for platform choice should also be stated clearly. Long-term flow through human intestinal organoids provides a concrete methodological anchor for such designs [46].

As studies move toward decision support, reproducibility is increasingly treated as a formal design requirement. Community recommendations emphasize fit-for-purpose quality management, explicit performance criteria, and reproducibility monitoring for microphysiological systems [81,82]. Standardization roadmaps also identify priorities such as terminology, materials, and system-level performance concepts. These elements support portability across platforms and laboratories [81,82]. A broader perspective on OoC capabilities and adoption challenges further supports positioning OoC as a tool selected to answer specific causal questions, rather than as an automatic “higher-relevance” upgrade [97].

## 7. Conclusions and Outlook: State of the Science, Limitations, and What Will Move the Needle

Human organoids (including ODMs) and OoC/MPS already enable mechanism-anchored biotoxin assessment by combining human-relevant tissue organization with functional endpoints aligned to key toxicodynamic outcomes. These include secretion, barrier/transport, hepatobiliary/renal function, and neural network activity. The most convincing studies make exposure geometry interpretable. They distinguish apical from basolateral and luminal from vascular exposure. They also connect toxin effects to the model’s critical determinants of internal dose and susceptibility. This is consistent with fit-for-purpose good-practice concepts for in vitro methods [32,33].

At the same time, the field is not yet uniformly “decision-ready.” The main limitations are uneven rigor in dosimetry fidelity, fit-for-purpose biological qualification and endpoint harmonization [32,33]. Dosimetry remains a major issue because nominal exposure can differ from delivered exposure. Biological qualification is also uneven, especially for polarity, transporter or enzyme competence, and functional stability during exposure. In addition, endpoints and acceptance criteria are not yet harmonized across laboratories and devices. Material interactions and compound loss are not unique to OoC/MPS. In both conventional organoid/ODM culture and OoC/MPS, adsorption or binding to plastics, ECM domes or hydrogels, surface coatings, and device materials can bias delivered exposure. This can distort nominal dose–response relationships unless these effects are measured, mitigated, or transparently bounded [49,50]. Many toxin studies also still rely on acute single-agent paradigms. When exposure scenarios are not well aligned to plausible human exposure, and when reporting does not support concentration–response interpretation, translation to public-health interpretation remains uncertain even when the underlying mechanism is clear [83].

What will most effectively move the needle are cross-cutting “infrastructure upgrades” that make results comparable, auditable, and reusable across groups. For biotoxin applications, these priorities should be explicit and platform-specific. In OoC/MPS, dosimetry fidelity should come first. Material interactions and flow can shift delivered exposure away from nominal values. Fit-for-purpose biological qualification should come next. Harmonization of a minimal functional endpoint core and acceptance criteria should then follow. Metadata and reproducibility checkpoints are also needed for reuse across laboratories. In organoids/ODMs, the order is slightly different. Biological qualification and exposure geometry or polarity control should come first. Endpoint harmonization and broader reproducibility infrastructure should follow after that. This ordering is consistent with current MPS quality and reproducibility frameworks. Quality-managed workflows with explicit performance criteria and reproducibility monitoring are increasingly viewed as the bridge from academic demonstrations to transferable evidence for MPS [81,82]. Reporting expectations are tightening through proposed minimum reporting elements designed to reduce irreproducibility driven by incomplete methods reporting across in vitro systems [80].

Accordingly, the most informative near-term studies in biotoxin assessment are likely to be comparative, quantitative, and explicitly route- and kinetics-aware. These include donor-diverse potency panels that apply harmonized functional endpoints across toxin classes and platforms. They also include escalation to coupled OoC designs only when coupling changes interpretation. In addition, chronic low-dose and mixture protocols should incorporate mechanistically justified context modifiers and should document delivered exposure over time. These studies should also report effect levels in model-agnostic terms, such as EC_x/benchmark responses. They should preserve QIVIVE-ready metadata and dosimetry. Under these conditions, organoid, ODM, and OoC evidence can more reliably progress from mechanistic proof-of-concept toward transparent decision support for public-health and regulatory toxicology [83].

## Figures and Tables

**Table 2:** Minimum reporting and qualification checklist for interpretable biotoxin studies using organoids/ODMs and OoC/MPS.

Determinant	Minimum to Report (Checklist)	Practical Notes/Examples	Where Discussed in This Review
Question & platform tier	Biotoxin(s), intended decision (e.g., mechanism, potency ranking, route effect, or QIVIVE readiness), platform format (3D organoid, ODM, OoC/MPS), and rationale for added complexity.	Complexity is not an inherent strength. A well-qualified ODM may be more informative than an under-characterized organoid. Use perfused/coupled OoC/MPS only when kinetics, perfusion, or organ–organ coupling is expected to change interpretation.	Section 2; Section 3.3; Section 6.1; Section 6.4 and Section 7
Biological source & model definition	Tissue region; donor characteristics or cell line/hiPSC line; species; differentiation stage; passage/expansion conditions; ECM/coating context.	Report donor *n* and distinguish biological from technical replicates. For hiPSC models, report the line and key phenotype markers.	Section 2; Section 3.2; Section 4.5 and Section 6.4
Fit-for-purpose biological qualification	Evidence of essential model competence under assay conditions (e.g., polarity, barrier integrity, lineage markers, relevant transporters/receptors, functional stability).	Enterotoxins: Polarity + CFTR/GC-C competence. Nephrotoxins: Transporter competence if uptake-limited. Neurotoxins: Functional electrophysiology, not morphology alone.	Section 4.1; Section 6.1; Section 6.3 and Section 7
Exposure geometry & kinetics	Route (apical/luminal vs. basolateral/vascular), delivery mode, exposure duration, sampling times, and infection model versus purified toxin.	In closed 3D organoids, specify whether exposure is external or luminal. In OoC/MPS, report flow and the causal role of kinetic control.	Section 4.2; Section 5.1, Section 5.2 and Section 5.3; Section 6.2 and Section 6.4
Dosimetry, matrix & material interactions	Nominal dose and dosing matrix; delivered concentration where measured; recovery/mass balance; free concentration when relevant; interactions with plastics, ECM/hydrogels, coatings, and device materials.	This is a cross-platform dosimetry issue, not a chip-specific limitation. If delivered exposure is not measured, state this clearly and interpret results as nominal dose-anchored.	Section 4.3; Section 6.2 and Section 7
Core functional endpoints (minimum comparable set)	A prespecified shared core of 3–5 endpoints, including: viability/cell injury; barrier/transport function (where relevant); a limited inflammatory panel (when relevant); and one organ-level functional readout linked to dominant pathophysiology.	Add toxin-specific extensions only when they change interpretation (e.g., transporter perturbation for OTA uptake dependence; pathway-linked second messengers for CT/STa).	Section 4.4; Section 5.1, Section 5.2 and Section 5.3; Section 6.3 and Section 7
Controls & assay acceptance criteria	Vehicle/negative controls; mechanism-anchoring positive controls; pathway inhibitors/antagonists where relevant; predefined acceptance criteria.	Report baseline stability before dosing; mechanistic claims should be supported by pathway-anchoring controls.	Section 4.4; Section 6.3 and Section 7
Replicates, statistics & effect metrics	Biological versus technical replicates; donor *n*; independent experiments; analysis plan; effect sizes and uncertainty.	Prefer concentration–response metrics and time dependence, not only significance testing.	Section 4.5; Section 6.4 and Section 7
Reproducibility & metadata for reuse	Key culture/device metadata, including materials, coatings, flow settings, sampling plan, and QC checkpoints.	Report enough metadata to separate biological heterogeneity from technical variability.	Section 4.5; Section 6.4 and Section 7

**Abbreviations:** CFTR, cystic fibrosis transmembrane conductance regulator; CT, cholera toxin; ECM, extracellular matrix; GC-C, guanylate cyclase-C; hiPSC, human induced pluripotent stem cell; ODM, organoid-derived monolayer; OoC, organ-on-chip; MPS, microphysiological systems; OTA, ochratoxin A; QIVIVE, quantitative in vitro–in vivo extrapolation; QC, quality control; STa, heat-stable enterotoxin.

## Data Availability

No new data were created or analyzed in this study.

## References

[1] Christopher W., Carly B., Ken T. (2022). The manipulation of cell signaling and host cell biology by cholera toxin. Cell. Signal..

[2] Wang H.X., Zhong Z.F., Luo Y., Cox E., Devriendt B. (2019). Heat-Stable Enterotoxins of Enterotoxigenic *Escherichia coli* and Their Impact on Host Immunity. Toxins.

[3] Biernbaum E.N., Kudva I.T. (2022). AB5 Enterotoxin-Mediated Pathogenesis: Perspectives Gleaned from Shiga Toxins. Toxins.

[4] Mycotoxins. World Health Organization, 2023. https://www.who.int/news-room/fact-sheets/detail/mycotoxins.

[5] Fletcher M.T., Netzel G. (2020). Food Safety and Natural Toxins. Toxins.

[6] Natural Toxins in Food. World Health Organization, 2023. https://www.who.int/news-room/fact-sheets/detail/natural-toxins-in-food.

[7] Cyanobacterial Toxins: Microcystins. World Health Organization, 2020. https://cdn.who.int/media/docs/default-source/wash-documents/wash-chemicals/microcystins-background-201223.pdf?sfvrsn=6d60aa6d_3.

[8] Multi-Country Outbreak of Cholera, External Situation Report #31—29 October 2025. World Health Organization 2025. https://www.who.int/publications/m/item/multi-country-outbreak-of-cholera--external-situation-report--31--29-october-2025.

[9] Trevejo R.T., Hunter M., Gilbreath T., Walton M., Bancroft J.E., DeBess E.E., Tran D., Cieslak P.R. (2025). Paralytic Shellfish Poisoning Outbreak-Oregon, United States, 2024. J. Food Prot..

[10] Chorus I., Welker M. (2021). Toxic Cyanobacteria in Water: A Guide to Their Public Health Consequences, Monitoring and Management.

[11] Zhang W.Q., Fujii N., Naren A.P. (2012). Recent advances and new perspectives in targeting CFTR for therapy of cystic fibrosis and enterotoxin-induced secretory diarrheas. Future Med. Chem..

[12] Roshni R., Siddharth R., Raj K. (2025). Microcystin-LR and its health impacts: Chemistry, transmission routes, mechanisms of toxicity and target organs. Toxicol. Rep..

[13] Imaoka T., Yang J., Wang L., McDonald M.G., Afsharinejad Z., Bammler T.K., Van Ness K., Yeung C.K., Rettie A.E., Himmelfarb J. (2020). Microphysiological system modeling of ochratoxin A-associated nephrotoxicity. Toxicology.

[14] Food Poisoning from Marine Toxins. Centers for Disease Control and Prevention, 2025. https://www.cdc.gov/yellow-book/hcp/environmental-hazards-risks/food-poisoning-from-marine-toxins.html.

[15] Porter R.J., Murray G.I., McLean M.H. (2020). Current concepts in tumour-derived organoids. Br. J. Cancer.

[16] Ma X.Y., Sun J.D., Ye Y.L., Ji J., Sun X.L. (2022). Application of triple co-cultured cell spheroid model for exploring hepatotoxicity and metabolic pathway of AFB1. Sci. Total Environ..

[17] Taroncher M., Gonzalez-Suarez A.M., Gwon K., Romero S., Reyes-Figueroa A.D., Rodríguez-Carrasco Y., Ruiz M.J., Stybayeva G., Revzin A., de Hoyos-Vega J.M. (2024). Using Microfluidic Hepatic Spheroid Cultures to Assess Liver Toxicity of T-2 Mycotoxin. Cells.

[18] Musah S., Bhattacharya R., Himmelfarb J. (2024). Kidney Disease Modeling with Organoids and Organs-on-Chips. Annu. Rev. Biomed. Eng..

[19] (2025). Roadmap to Reducing Animal Testing in Preclinical Safety Studies. U.S. Food and Drug Administration. https://www.fda.gov/files/newsroom/published/roadmap_to_reducing_animal_testing_in_preclinical_safety_studies.pdf.

[20] New Approach Methods (NAMs) Work Plan. U.S. Environmental Protection Agency 2021. https://www.epa.gov/system/files/documents/2021-11/nams-work-plan_11_15_21_508-tagged.pdf.

[21] Administrator Zeldin Gets EPA Back on Track to Eliminate Animal Testing After Biden Admin Halted Phase Out. U.S. Environmental Protection Agency, 2026. https://www.epa.gov/newsreleases/administrator-zeldin-gets-epa-back-track-eliminate-animal-testing-after-biden-admin.

[22] New Approach Methodologies (NAMs) in Future Food Safety Risk Assessment. World Health Organization, 2025. https://www.who.int/news-room/events/detail/2025/06/18/default-calendar/new-approach-methodologies-%28nams%29-in-future-food-safety-risk-assessment.

[23] Roodsant T., Navis M., Aknouch I., Renes I.B., van Elburg R.M., Pajkrt D., Wolthers K.C., Schultsz C., van der Ark K.C.H., Sridhar A. (2020). A Human 2D Primary Organoid-Derived Epithelial Monolayer Model to Study Host-Pathogen Interaction in the Small Intestine. Front. Cell. Infect. Microbiol..

[24] Bhatia S.N., Ingber D.E. (2014). Microfluidic organs-on-chips. Nat. Biotechnol..

[25] Toepke M.W., Beebe D.J. (2006). PDMS absorption of small molecules and consequences in microfluidic applications. Lab Chip.

[26] Zomer-van Ommen D.D., Pukin A.V., Fu O., Van Ufford L., Janssens H.M., Beekman J.M., Pieters R.J. (2016). Functional Characterization of Cholera Toxin Inhibitors Using Human Intestinal Organoids. J. Med. Chem..

[27] Pattison A.M., Blomain E.S., Merlino D.J., Wang F., Crissey M.A.S., Kraft C.L., Rappaport J.A., Snook A.E., Lynch J.P., Waldman S.A. (2016). Intestinal Enteroids Model Guanylate Cyclase C-Dependent Secretion Induced by Heat-Stable Enterotoxins. Infect. Immun..

[28] Lee Y., Kim M.H., Alves D.R., Kim S., Lee L.P., Sung J.H., Park S. (2021). Gut-Kidney Axis on Chip for Studying Effects of Antibiotics on Risk of Hemolytic Uremic Syndrome by Shiga Toxin-Producing *Escherichia coli*. Toxins.

[29] Yue H.-B., Sivasankaran M.S., Ottakandathil B.R., Wu Z.-L., So M.-T., Chung H.-Y., Wong K.-Y., Kwong-Hang T., Chi-Hang L. (2024). Environmental Toxin Biliatresone-Induced Biliary Atresia-like Abnormal Cilia and Bile Duct Cell Development of Human Liver Organoids. Toxins.

[30] Carstens K.E., Gronskaya E., Jaeckel D., Bertoli J., Cuevas K.R., Dorier J., Wang S., Lopez-Rodriguez D., Shafer T.J., Zurich M.G. (2025). Application of a high-density microelectrode array assay using a 3D human iPSC-derived brain microphysiological system model for in vitro neurotoxicity screening of environmental compounds. Arch. Toxicol..

[31] Pedrosa C.D.G., Souza L.R.Q., Gomes T.A., de Lima C.V.F., Ledur P.F., Karmirian K., Barbeito-Andres J., Costa M.D., Higa L.M., Rossi A.D. (2020). The cyanobacterial saxitoxin exacerbates neural cell death and brain malformations induced by Zika virus. PLoS Neglected Trop. Dis..

[32] Organisation for Economic Co-operation and Development (OECD) (2018). Guidance Document on Good In Vitro Method Practices (GIVIMP).

[33] Pamies D., Leist M., Coecke S., Bowe G., Allen D.G., Gstraunthaler G., Bal-Price A., Pistollato F., de Vries R.B.M., Hogberg H.T. (2022). Guidance Document on Good Cell and Tissue Culture Practice 2.0 (GCCP 2.0). Altex-Altern. Anim. Exp..

[34] Achilli T.M., Meyer J., Morgan J.R. (2012). Advances in the formation, use and understanding of multi-cellular spheroids. Expert Opin. Biol. Ther..

[35] Kang S.M., Kim D., Lee J.H., Takayama S., Park J.Y. (2021). Engineered Microsystems for Spheroid and Organoid Studies. Adv. Healthc. Mater..

[36] Kretzschmar K., Clevers H. (2016). Organoids: Modeling Development and the Stem Cell Niche in a Dish. Dev. Cell.

[37] Sato T., Vries R.G., Snippert H.J., van de Wetering M., Barker N., Stange D.E., van Es J.H., Abo A., Kujala P., Peters P.J. (2009). Single Lgr5 stem cells build crypt-villus structures in vitro without a mesenchymal niche. Nature.

[38] Kakni P., López-Iglesias C., Truckenmüller R., Habibovic P., Giselbrecht S. (2023). PSC-derived intestinal organoids with apical-out orientation as a tool to study nutrient uptake, drug absorption and metabolism. Front. Mol. Biosci..

[39] Aisenbrey E.A., Murphy W.L. (2020). Synthetic alternatives to Matrigel. Nat. Rev. Mater..

[40] Lancaster M.A., Renner M., Martin C.A., Wenzel D., Bicknell L.S., Hurles M.E., Homfray T., Penninger J.M., Jackson A.P., Knoblich J.A. (2013). Cerebral organoids model human brain development and microcephaly. Nature.

[41] Pettinato G., Wen X.J., Zhang N. (2014). Formation of Well-defined Embryoid Bodies from Dissociated Human Induced Pluripotent Stem Cells using Microfabricated Cell-repellent Microwell Arrays. Sci. Rep..

[42] van Dooremalen W.T.M., Derksen M., Roos J.L., Barón C.H., Verissimo C.S., Vries R.G.J., Boj S.F., Pourfarzad F. (2021). Organoid-Derived Epithelial Monolayer: A Clinically Relevant In Vitro Model for Intestinal Barrier Function. J. Vis. Exp..

[43] Leung C.M., de Haan P., Ronaldson-Bouchard K., Kim G.A., Ko J., Rho H.S., Chen Z., Habibovic P., Li Jeon N., Takayama S. (2022). A guide to the organ-on-a-chip. Nat. Rev. Methods Primers.

[44] (2024). Food Safety. World Health Organization. https://www.who.int/news-room/fact-sheets/detail/food-safety.

[45] Wang L., Han J.L., Su W.G., Li A.Q., Zhang W.X., Li H.M., Hu H.L., Song W., Xu C.H., Chen J. (2023). Gut-on-a-chip for exploring the transport mechanism of Hg(II). Microsyst. Nanoeng..

[46] Sidar B., Jenkins B.R., Huang S., Spence J.R., Walk S.T., Wilking J.N. (2019). Long-term flow through human intestinal organoids with the gut organoid flow chip (GOFlowChip). Lab Chip.

[47] Co J.Y., Margalef-Català M., Monack D.M., Amieva M.R. (2021). Controlling the polarity of human gastrointestinal organoids to investigate epithelial biology and infectious diseases. Nat. Protoc..

[48] Choi J.H., Loarca L., De Hoyos-Vega J.M., Dadgar N., Loutherback K., Shah V.H., Stybayeva G., Revzin A. (2020). Microfluidic confinement enhances phenotype and function of hepatocyte spheroids. Am. J. Physiol.-Cell Physiol..

[49] van Meer B.J., de Vries H., Firth K.S.A., van Weerd J., Tertoolen L.G.J., Karperien H.B.J., Jonkheijm P., Denning C., Ijzerman A.P., Mummery C.L. (2017). Small molecule absorption by PDMS in the context of drug response bioassays. Biochem. Biophys. Res. Commun..

[50] Hwang J., Sullivan M.O., Kiick K.L. (2020). Targeted Drug Delivery via the Use of ECM-Mimetic Materials. Front. Bioeng. Biotechnol..

[51] Shirure V.S., George S.C. (2017). Design considerations to minimize the impact of drug absorption in polymer-based organ-on-achip platforms. Lab Chip.

[52] Pöschl F., Höher T., Pirklbauer S., Wolinski H., Lienhart L., Ressler M., Riederer M. (2023). Dose and route dependent effects of the mycotoxin deoxynivalenol in a 3D gut-on-a-chip model with flow. Toxicol. Vitr..

[53] Dekkers J.F., Wiegerinck C.L., de Jonge H.R., Bronsveld I., Janssens H.M., de Winter-de Groot K.M., Brandsma A.M., de Jong N.W.M., Bijvelds M.J.C., Scholte B.J. (2013). A functional CFTR assay using primary cystic fibrosis intestinal organoids. Nat. Med..

[54] Singla A., Boucher A., Wallom K.L., Lebens M., Kohler J.J., Platt F.M., Yrlid U. (2023). Cholera intoxication of human enteroids reveals interplay between decoy and functional glycoconjugate ligands. Glycobiology.

[55] Hoffmann P., Schnepel N., Langeheine M., Künnemann K., Grassl G.A., Brehm R., Seeger B., Mazzuoli-Weber G., Breves G. (2021). Intestinal organoid-based 2D monolayers mimic physiological and pathophysiological properties of the pig intestine. PLoS ONE.

[56] Joo S.S., Gu B.H., Park Y.J., Rim C.Y., Kim M.J., Kim S.H., Cho J.H., Kim H.B., Kim M. (2022). Porcine Intestinal Apical-Out Organoid Model for Gut Function Study. Animals.

[57] Yibcharoenporn C., Kongkaew T., Worakajit N., Khumjiang R., Saetang P., Satitsri S., Rukachaisirikul V., Muanprasat C. (2024). Inhibition of CFTR-mediated intestinal chloride secretion by nornidulin: Cellular mechanisms and anti-secretory efficacy in human intestinal epithelial cells and human colonoids. PLoS ONE.

[58] Lopez A.V., Waddell A., Antonacci S., Castillo D., Santucci N., Ollberding N.J., Eshleman E.M., Denson L.A., Alenghat T. (2023). Microbiota-derived butyrate dampens linaclotide stimulation of the guanylate cyclase C pathway in patient-derived colonoids. Neurogastroenterol. Motil..

[59] Motyka N.I., Stewart S.R., Hollifield I.E., Kyllo T.R., Mansfield J.A., Norton E.B., Clements J.D., Bitoun J.P. (2021). Elevated Extracellular cGMP Produced after Exposure to Enterotoxigenic *Escherichia coli* Heat-Stable Toxin Induces Epithelial IL-33 Release and Alters Intestinal Immunity. Infect. Immun..

[60] Park S.E., Georgescu A., Huh D. (2019). Organoids-on-a-chip. Science.

[61] Karve S.S., Pradhan S., Ward D.V., Weiss A.A. (2017). Intestinal organoids model human responses to infection by commensal and Shiga toxin producing *Escherichia coli*. PLoS ONE.

[62] Pradhan S., Karve S.S., Weiss A.A., Hawkins J., Poling H.M., Helmrath M.A., Wells J.M., McCauley H.A. (2020). Tissue Responses to Shiga Toxin in Human Intestinal Organoids. Cell. Mol. Gastroenterol. Hepatol..

[63] Tovaglieri A., Sontheimer-Phelps A., Geirnaert A., Prantil-Baun R., Camacho D.M., Chou D.B., Jalili-Firoozinezhad S., de Wouters T., Kasendra M., Super M. (2019). Species-specific enhancement of enterohemorrhagic *E. coli* pathogenesis mediated by microbiome metabolites. Microbiome.

[64] Garcia A.L.C., Kucab J.E., Al-Serori H., Beck R.S.S., Bellamri M., Turesky R.J., Groopman J.D., Francies H.E., Garnett M.J., Huch M. (2024). Tissue Organoid Cultures Metabolize Dietary Carcinogens Proficiently and Are Effective Models for DNA Adduct Formation. Chem. Res. Toxicol..

[65] Hanyu H., Yokoi Y., Nakamura K., Ayabe T., Tanaka K., Uno K., Miyajima K., Saito Y., Iwatsuki K., Shimizu M. (2020). Mycotoxin Deoxynivalenol Has Different Impacts on Intestinal Barrier and Stem Cells by Its Route of Exposure. Toxins.

[66] Lee M.G., Lee B.R., Lee P., Choi S., Kim J.H., Oh M.H., Yoo J.G. (2024). Apical-out intestinal organoids as an alternative model for evaluating deoxynivalenol toxicity and Lactobacillus detoxification in bovine. Sci. Rep..

[67] Bova R.A., Lamont A.C., Picou T.J., Ho V.B., Gilchrist K.H., Melton-Celsa A.R. (2023). Shiga Toxin (Stx) Type 1a and Stx2a Translocate through a Three-Layer Intestinal Model. Toxins.

[68] Vedoy O.B., Steinsland H., Sakkestad S.T., Sommerfelt H., Hanevik K. (2023). Strong Association between Diarrhea and Concentration of Enterotoxigenic *Escherichia coli* Strain TW10722 in Stools of Experimentally Infected Volunteers. Pathogens.

[69] Cornick N.A., Jelacic S., Ciol M.A., Tarr P.I. (2002). *Escherichia coli* O157:H7 infections: Discordance between filterable fecal Shiga toxin and disease outcome. J. Infect. Dis..

[70] Bovard D., Sandoz A., Luettich K., Frentzel S., Iskandar A., Marescotti D., Trivedi K., Guedj E., Dutertre Q., Peitsch M.C. (2018). A lung/liver-on-a-chip platform for acute and chronic toxicity studies. Lab Chip.

[71] Kanabekova P., Kadyrova A., Kulsharova G. (2022). Microfluidic Organ-on-a-Chip Devices for Liver Disease Modeling In Vitro. Micromachines.

[72] Chen W.Y., Evangelista E.A., Yang J.D., Kelly E.J., Yeung C.K. (2021). Kidney Organoid and Microphysiological Kidney Chip Models to Accelerate Drug Development and Reduce Animal Testing. Front. Pharmacol..

[73] Zingales V., Piunti C., Micheli S., Cimetta E., Ruiz M.J. (2024). Development of an Easy-To-Use Microfluidic System to Assess Dynamic Exposure to Mycotoxins in 3D Culture Models: Evaluation of Ochratoxin A and Patulin Cytotoxicity. Foods.

[74] Huo W.Y., Qiao Y.Y., He X.R., Wang C.L., Li R.Q., Che L., Li E.K. (2025). Mycotoxins and the Intestinal Epithelium: From Barrier Injury to Stem Cell Dysfunction. Toxins.

[75] Schwartz-Zimmermann H.E., Hametner C., Nagl V., Fiby I., Macheiner L., Winkler J., Dänicke S., Clark E., Pestka J.J., Berthiller F. (2017). Glucuronidation of deoxynivalenol (DON) by different animal species: Identification of iso-DON glucuronides and iso-deepoxy-DON glucuronides as novel DON metabolites in pigs, rats, mice, and cows. Arch. Toxicol..

[76] Jin J., Spenkelink A., Beekmann K., Baccaro M., Xing F.G., Rietjens I. (2021). Species Differences in in vitro and Estimated in vivo Kinetics for Intestinal Microbiota Mediated Metabolism of Acetyl-deoxynivalenols. Mol. Nutr. Food Res..

[77] Waisbourd-Zinman O., Koh H., Tsai S.N., Lavrut P.M., Dang C., Zhao X., Pack M., Cave J., Hawes M., Koo K.A. (2016). The toxin biliatresone causes mouse extrahepatic cholangiocyte damage and fibrosis through decreased glutathione and SOX17. Hepatology.

[78] Fried S., Gilboa D., Har-Zahav A., Lavrut P.M., Du Y., Karjoo S., Russo P., Shamir R., Wells R.G., Waisbourd-Zinman O. (2020). Extrahepatic cholangiocyte obstruction is mediated by decreased glutathione, Wnt and Notch signaling pathways in a toxic model of biliary atresia. Sci. Rep..

[79] Palumbo R., Crisci A., Venâncio A., Abrahantes J.C., Dorne J.L., Battilani P., Toscano P. (2020). Occurrence and Co-Occurrence of Mycotoxins in Cereal-Based Feed and Food. Microorganisms.

[80] Mohapatra R., Leist M., von Aulock S., Hartung T. (2025). Guidance for Good In Vitro Reporting Standards (GIVReSt)-A Draft for Stakeholder Discussion and Background Documentation. Altex-Altern. Anim. Exp..

[81] Pamies D., Ekert J., Zurich M.G., Frey O., Werner S., Piergiovanni M., Freedman B.S., Teo A.K.K., Erfurth H., Reyes D.R. (2024). Recommendations on fit-for-purpose criteria to establish quality management for microphysiological systems and for monitoring their reproducibility. Stem Cell Rep..

[82] Miedel M.T., Mahboubeh V., Mengying X., Brooks M.M., Gavlockc D.C., Celeste R., Jaideep B., Alejandro S.-G., Gough A., Lansing Taylor D. (2025). Validation of microphysiological systems for interpreting patient heterogeneity requires robust reproducibility analytics and experimental metadata. Cell Rep. Methods.

[83] Chang X.Q., Tan Y.M., Allen D.G., Bell S., Brown P.C., Browning L., Ceger P., Gearhart J., Hakkinen P.J., Kabadi S.V. (2022). IVIVE: Facilitating the Use of In Vitro Toxicity Data in Risk Assessment and Decision Making. Toxics.

[84] Zingales V., Esposito M.R., Torriero N., Taroncher M., Cimetta E., Ruiz M.J. (2023). The Growing Importance of Three-Dimensional Models and Microphysiological Systems in the Assessment of Mycotoxin Toxicity. Toxins.

[85] Chowdhury R.R., Rose S., Ezan F., Sovadinová I., Babica P., Langouët S. (2024). Hepatotoxicity of cyanotoxin microcystin-LR in human: Insights into mechanisms of action in the 3D culture model Hepoid-HepaRG. Environ. Pollut..

[86] Basu A., Dydowiczová A., Ctverácková L., Jasa L., Trosko J.E., Bláha L., Babica P. (2018). Assessment of Hepatotoxic Potential of Cyanobacterial Toxins Using 3D In Vitro Model of Adult Human Liver Stem Cells. Environ. Sci. Technol..

[87] Hercog K., Stampar M., Stern A., Filipic M., Zegura B. (2020). Application of advanced HepG2 3D cell model for studying genotoxic activity of cyanobacterial toxin cylindrospermopsin. Environ. Pollut..

[88] Chowdhury R.R., Grosso M.F., Gadara D.C., Spácil Z., Vidová V., Sovadinova I., Babica P. (2024). Cyanotoxin cylindrospermopsin disrupts lipid homeostasis and metabolism in a 3D in vitro model of the human liver. Chem.-Biol. Interact..

[89] Fischer W.J., Altheimer S., Cattori V., Meier P.J., Dietrich D.R., Hagenbuch B. (2005). Organic anion transporting polypeptides expressed in liver and brain mediate uptake of microcystin. Toxicol. Appl. Pharmacol..

[90] Kinnear S. (2010). Cylindrospermopsin: A Decade of Progress on Bioaccumulation Research. Mar. Drugs.

[91] Froscio S.M., Humpage A.R., Burcham P.C., Falconer I.R. (2003). Cylindrospermopsin-induced protein synthesis inhibition and its dissociation from acute toxicity in mouse hepatocytes. Environ. Toxicol..

[92] Humpage A.R., Fontaine F., Froscio S., Burcham P., Falconer I.R. (2005). Cylindrospermopsin genotoxicity and cytotoxicity: Role of cytochrome P-450 and oxidative stress. J. Toxicol. Environ. Health-Part A-Curr. Issues.

[93] Kittler K., Hurtaud-Pessel D., Maul R., Kolrep F., Fessard V. (2016). In vitro metabolism of the cyanotoxin cylindrospermopsin in HepaRG cells and liver tissue fractions. Toxicon.

[94] Paguirigan A.L., Beebe D.J. (2009). From the cellular perspective: Exploring differences in the cellular baseline in macroscale and microfluidic cultures. Integr. Biol..

[95] Haque A., Gheibi P., Gao Y.D., Foster E., Son K.J., You J., Stybayeva G., Patel D., Revzin A. (2016). Cell biology is different in small volumes: Endogenous signals shape phenotype of primary hepatocytes cultured in microfluidic channels. Sci. Rep..

[96] Kim H.J., Li H., Collins J.J., Ingber D.E. (2016). Contributions of microbiome and mechanical deformation to intestinal bacterial overgrowth and inflammation in a human gut-on-a-chip. Proc. Natl. Acad. Sci. USA.

[97] Ingber D.E. (2022). Human organs-on-chips for disease modelling, drug development and personalized medicine. Nat. Rev. Genet..

